# A gut (microbiome) feeling about addiction: Interactions with stress and social systems

**DOI:** 10.1016/j.ynstr.2024.100629

**Published:** 2024-03-18

**Authors:** Rubén García-Cabrerizo, John F. Cryan

**Affiliations:** a IUNICS, University of the Balearic Islands, Palma, Spain; b Health Research Institute of the Balearic Islands (IdISBa), Palma, Spain; c Department of Medicine, University of the Balearic Islands, Palma, Spain; d APC Microbiome Ireland, University College Cork, Cork, Ireland; e Department of Anatomy and Neuroscience, University College Cork, Cork, Ireland

**Keywords:** Microbiota, Stress, Social behavior, Drug addiction

## Abstract

In recent years, an increasing attention has given to the intricate and diverse connection of microorganisms residing in our gut and their impact on brain health and central nervous system disease. There has been a shift in mindset to understand that drug addiction is not merely a condition that affects the brain, it is now being recognized as a disorder that also involves external factors such as the intestinal microbiota, which could influence vulnerability and the development of addictive behaviors. Furthermore, stress and social interactions, which are closely linked to the intestinal microbiota, are powerful modulators of addiction. This review delves into the mechanisms through which the microbiota-stress-immune axis may shape drug addiction and social behaviors. This work integrates preclinical and clinical evidence that demonstrate the bidirectional communication between stress, social behaviors, substance use disorders and the gut microbiota, suggesting that gut microbes might modulate social stress having a significance in drug addiction.

## 1 Introduction

Drug addiction is considered a chronic relapsing disorder characterized by compulsive drug seeking and taking behaviors despite the negative consequences. In the last decades, its impact has taken on an epidemic dimension, with approximately 300 million people having consumed some form of illicit drug at least once in the past year, and nearly 40 million people affected by substance use disorders (UNODC, 2023). This has led to devastating consequences for public health, economic and social wellbeing, currently resulted in over 600,000 deaths annually worldwide each year. Furthermore, reports from the National Survey on Drug Use and Health (NSDUH) of the US reveal that among individuals aged 12 and above, 59.8% reported recent use of illicit drugs in the last month, suggesting a widespread use among the population. Despite the great advances achieved in understanding the neurobiology of drug addiction, current treatments (i.e., psychosocial and/or pharmacology interventions) has a relapse rate of 50–70% (Brandon et al., 2007; Lappan et al., 2020), highlighting the urgency of new therapeutic approaches to overcome the shortcomings of the present drug addiction treatments.

An extensive literature has shown that drugs of abuse affect systems that govern the reward pathways, learning, memory processes, emotion and cognitive functions (Kalivas and O’Brien, 2008; Koob and Volkow, 2010; Polter and Kauer, 2014; Russo and Nestler, 2013). The pleasurable effects of addictive substances are linked to their impact on the mesocorticolimbic system, which subsequently affects behaviors related to motivation, emotions, and feelings (Feltenstein and See, 2008). In particular, changes in how the brain processes rewards are considered a pivotal factor in the progression of addiction, and several factors such as stress, social behaviors and more recently the gut microbiota have been proposed as modulators of the brain reward systems (El Rawas et al., 2020; García-Cabrerizo et al., 2021; Koob and Schulkin, 2019). Despite the wide range of behavioral effects and distinct pharmacological characteristics exhibited by various drugs of abuse, a unifying aspect among them is the enhanced mesocorticolimbic dopamine activity. This neural pathway has been extensively associated with processing rewarding stimuli, encompassing both natural and non-natural rewards like addictive substances (Olsen, 2011). Stressful events may negatively regulate the dopaminergic reward system, alerting reward sensitivity and suggesting that this dopaminergic system is necessary for coping with these stress-related behaviors (Bloomfield et al., 2019; Finlay and Zigmond, 1997; Thierry et al., 1976). Given the close relationship between the brain systems involved in addiction and stress, environmental stressors can lead to a long-term change in the function of the brain reward system (Montagud-Romero et al., 2018). Furthermore, stressors and drug administration activate central and peripheral stress pathways, promoting inflammatory responses in the brain, and these connections are pertinent as immune activity regulates mood and behaviors (Wohleb et al., 2014). The innate immune system serves as the first line of defense against infection and stressors, recruiting immune cells through the production of pro-inflammatory cytokines that may act on the brain inducing neuroinflammation. Increased neuroinflammation affects dopamine metabolism and produces a set of symptoms known as sickness behavior, including anhedonia, decreased mobility, cognitive impairment as well as loss of interest for physical and social environments (Dantzer et al., 2008; Miller et al., 2013). Recent evidence reveals that immune system and more specifically inflammatory processes are powerful regulators of neuronal circuits supporting social behaviors (Eisenberger et al., 2017), suggesting a co-evolutionary link between the immune response and social behaviors (Filiano et al., 2016). In this context, social factors are known to have important effects on behavioral responses to stress, because social interactions can be a powerful source of stress, promoting substance use disorders (Bardo et al., 2021). Social and demographic changes have led to an increased prevalence of loneliness and social isolation as well as overcrowding and competitivity for opportunity and resources in modern societies, promoting social stress exposure. Furthermore, prolonged psychosocial stressor, such as being abused by a partner, living in a socially unstable environment or exposure to bullying or harassment, is a risk factor to develop substance use disorders (Kirsch and Lippard, 2022; Vrijen et al., 2021). In this line, substance use is frequently associated with social self-isolation, affecting social engagement in drug addicts, acting as an aversive stimulus and promoting drug intake to cope with this social stress. Nevertheless, social interactions can also protect individuals from an exaggerated physiological stress response to challenging situations, preventing the development of stress-related pathologies and drug addiction (Sandi and Haller, 2015; Venniro et al., 2018, 2019).

**Fig. 1:**
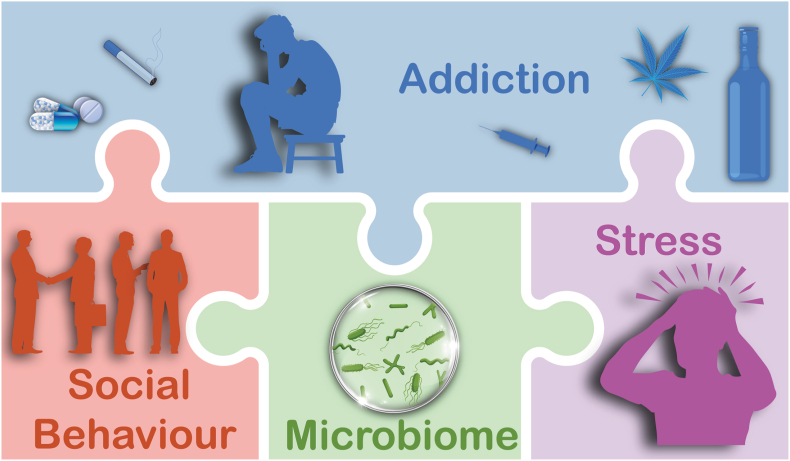
Drug addiction is a chronic relapsing disorder with the lack of effective treatments that emphasizing the need to expand therapeutic options. Dysregulations triggered by stressors, social factors, and gut microbiota may play a role in the development and progression of drug addiction.

Recently, the gut microbiota has emerged as a key regulator of brain reward processes, representing a potential target for intervention in drug addiction (García-Cabrerizo et al., 2021; Meckel and Kiraly, 2019). Preliminary works indicates that drugs of abuse induce alterations in the gut microbiota and that changes could be involved in development of substance use disorders (Acharya et al., 2017; Kirpich et al., 2008; Leclercq et al., 2014; Mutlu et al., 2009; Ning et al., 2017; Scorza et al., 2019; Volpe et al., 2014; Wang et al., 2012). Furthermore, gut microbes are essential for the correct programing of social behaviors, impacting sociability and its neurobiological underpinnings across the animal kingdom (Sherwin et al., 2019) and targeting the gut microbiota has been shown to modulate social responses (O'Connor et al., 2021; Sgritta et al., 2019). Great efforts are ongoing to understand the etiological factors that are responsible for the development of addiction in the hope that they can be exploited for therapeutic benefit. Three factors worth noting are stress and the hypothalamic-pituitary-adrenal (HPA) axis, social behaviors, and more recently the gut microbiome. In this review, we integrate data from both animal and human studies on all three of these factors in the context of their role in the etiology and development of addiction (Fig. 1).

## 2 Microbiota-gut-brain axis

The gastrointestinal tract hosts a diverse array of microorganisms, collectively called to as the microbiota, encompassing bacteria, *archaea*, yeasts, single-celled eukaryotes, helminth parasites and viruses called bacteriophages (Gilbert et al., 2018). Over the past years, the pace of microbiome research has surged, unveiling the numerous ways in which these tiny residents significantly influence our everyday existence. It has become evident that the microbiota plays a crucial role in shaping human health and disease, serving as a fundamental regulator of host physiology. In the human body, there are as many microbial cells as human cells, and in terms of genes possessing 100 times the genes in the human genome (Sender et al., 2016), exerting influence on various aspects of human physiology and biology through interactions with their host (Frank and Pace, 2008; Kurokawa et al., 2007). This mutual interaction benefits both parties, with microbes playing roles in energy regulation, gut barrier function, protection from pathogens and immune system function, while the host provides a conducive environment. The core composition of the adult gut bacteria, established in early life, is influenced by factors like delivery mode, breastfeeding, diet, medication, viral or bacterial infections, and stress (Borre et al., 2014b; Hamady and Knight, 2009). This critical development period aligns with the simultaneous growth and maturation of neurons in the young brain and a similar profile occurs in old age, where a decline in microbiota complexity parallels a decrease in neuronal complexity (Biagi et al., 2012; Borre et al., 2014a, 2014b). Collaborative projects have examined and characterized the gut microbiota on a population scale, categorizing prokaryote species in 11 different phyla such as *Proteobacteria*, *Firmicutes*, *Actinobacteria*, and *Bacteroidetes* constituting over 90% of the microbiome, while *Fusobacteria* and *Verrucomicrobia* phyla are present in limited abundance (Eckburg et al., 2005; Hugon et al., 2015; J. J. Li et al., 2014). When pathogenic bacteria escape the gut, possibly due to a compromised gut barrier resulting from exposure to drugs of abuse or stress, they can trigger a proinflammatory response. The cytokines produced in the gastrointestinal tract then travel to the brain through the bloodstream, activating the HPA axis, leading to the secretion of cortisol, which affects immune cell activity locally and systemically. Conversely, beneficial gut bacteria can stimulate the release of anti-inflammatory cytokines and produce microbial metabolites that enhance gut barrier integrity and immune balance while modulating cytokine production (Al Bander et al., 2020). These metabolites can cross the blood-brain barrier, influencing microglial maturation and psychological functioning through various pathways, including humoral/immune effects, interactions with histone deacetylases, G protein-coupled receptors, and hormonal pathways (Erny et al., 2015; O'Riordan et al., 2022).

## 3 Stress and the microbiome

### 3.1 Stress, immunity and the gut microbiota

Stress, immunity and gastrointestinal function are intricately linked (Rea et al., 2016; Solano and Menard, 2023). Accumulating evidence support the view that stressors can disrupt the permeability of the gut barrier, allowing bacteria and their components to cross the gut barrier activating the mucosal immune responses and perhaps activating the HPA axis (Leigh et al., 2023) (Table 1). In previous studies, it was observed that rats exposed to an acute stressor exhibit increased gut permeability (Kiliaan et al., 1998). Furthermore, other studies described a decrease in the gene expression of gut tight junction proteins and also an overproduction of INF-γ (Demaude et al., 2006). Neurochemicals related to stress, such as catecholamines, have the potential to promote growth of pathogenic strain of *Escherichia coli,* suggesting that stress mediators might create a proper environment to stimulate the proliferation of certain gut bacteria, contributing to the development of the stress response (Freestone et al., 2002). In this line, chronic stressors induced marked alteration in gut physiology and microbial composition in rodents. Chronic restraint stress has been linked to an increase in bacterial taxa associated with inflammatory processes in mice (Gao et al., 2018). Additionally, an increase in the relative abundance of *Akkermansia* in the gut was associated with higher INF-γ and TNF-α in the hippocampus of mice exposed to chronic unpredictable mild stress (Li et al., 2019).

**Table 1:** Key studies evaluating stress and the microbiota-gut-brain axis.

Type of stress	Species, strain	Sex/age	Microbiota modulation	Gut	Taxonomy	Function	Work
**Preclinical evidence**							
Acute stress	Rat, Wistar-Kyoto	Male/Adult	–	Stress increased gut permeability (jejunum)	–	–	Kiliaan et al. (1998)
Acute stress	Mice, Swiss 3T3	Male/8 weeks old	–	Stress increased gut permeability (colon)	–	Reduced gene expression of ZO-2 and occludin, overproduction INF-γ	Demaude et al. (2006)
Acute stress	Mice/Swiss Webster	Male and female/6–9 weeks	Germ-free	–	–	Increased corticosterone response and reductions in TNF-α in plasma	Clarke et al. (2013)
Acute restrain stress	Mice/C57BL6	Male/10–11 weeks	Germ-free	–	–	Changes in peripheral myeloid cells	van de Wouw et al. (2020)
Novel environment exposure	Rat/F334	Male/Adult	Germ-free	–	–	Increased in serum corticosterone in GF rats	Crumeyrolle-Arias et al. (2014)
Acute Forced-swim test	Mice/BALB/c	Male/Adult	*L. rhamnosus JB-1*	–	–	*L. rhamnosus* JB-1 reduced serum corticosterone	Bravo et al. (2011)
Water avoidance stress	Mice/C57BL6	Male/Adult	*B. longum* R0175 and *L. helveticus* R0052	–	–	*changes in peripheral myeloid cells*	Ait-Belgnaoui et al. (2014)
Partial restrain stress	Rat, Wistar	Female/adult	*L. farciminis*	–	–	*L. farciminis* suppressed stress-induced hyperpermeability, endotoxemia and prevented HPA axis stress response and neuroinflammation	Ait-Belgnaoui et al. (2012)
Chronic restraint stress	Mice, C57BL/6	Male/Adult	–	–	Increased *Helicobacter*, *Peptostreptococcaceae*, *Streptococcus*, and *Entero-coccus* faecalis, but decreased levels of *Rikenella*, *Roseburia*, and *Lachnospiracea*	Increased IL-6 in colon, increased behavioral despair	Gao et al. (2018)
Chronic unpredictable mild stress	Mice, C57BL/6	Male/7 weeks old	–	–	Low abundance *Lactobacillus* and higher abundance *Akkermansia*	Increased INF-γ and TNF-α in the hippocampus, decreased sucrose preference and increased behavioral despair	Li et al. (2019)
Restrain stress	Mice/BALB/c	Male/9 weeks	Germ-free	–	–	Adrenocorticotropic hormone and corticosterone elevation in germ-free mice	Sudo et al. (2004)
Chronic unpredictable stress	Mice/C57BL6	Male/7 weeks old	FOS and GOS	–	–	GOS and the FOS + GOS combination reduced stress-induced corticosterone release and pro-inflammatory cytokines.	Burokas et al. (2017)
Chronic mild stress	Rat/Wistar	Male/Adult	Fecal material transplant	–	–	Stress and fecal material transplant from stressed animals increased neuroinflammation in the hippocampus	Marcondes Ávila et al. (2020)
Chronic psychosocial stress	Mice/C57BL6	Male/Adult	Butyrate, acetate and propionate	Short-chain fatty acids reduced stress-induced increases in intestinal permeability	Neither stress nor short-chain fatty acids affected cecal microbiota diversity	Short-chain fatty acids reduced heightened stress-responsiveness	van de Wouw et al. (2018)
Unpredictable chronic mild stress	Mice/C57BL6	Male/8–10 weeks old	Fecal material transplant	–	Stress increased *Bacteroidetes* and decreased *Firmicutes* and *Verrucomicrobia*	Stress and FMT from stressed mice increased IL-6, TNF-α and IL-1β in hippocampus	Siopi et al. (2023)
Maternal separation	Rat, Sprague-Dawley	Male/PND 2 to 12	–	–	–	Increased INF-γ and TNF-α in blood	O'Mahony et al. (2009)
Maternal separation	Rat, Sprague-Dawley	Male/PND 2 to 12	–		Increased *Akkermansia* spp. and decreased *Rikenella* spp	Lower levels of corticosterone	Pusceddu et al. (2015)
Maternal separation	Rat, Sprague-Dawley	Male/PND 2 to 12 (offspring)	*B. infantis*	–	–	*B. infantis* attenuated pro-inflammatory immune and corticosterone response	Desbonnet et al. (2010)
Maternal separation	Rat, Sprague-Dawley	Male/adult (offspring)	*B. animales* and *P. jensenii*	–	–	Probiotic reduced serum corticosterone	Gareau et al. (2007)
Restrain pregnant dams	Rat, Sprague-Dawley	Male/Adult (offspring)	–	Deficit colon innervation	Decreased *Lactobacillus*, increased *Oscillobacter, Anaerotruncus* and *Peptococcus*	Increased HPA axis response to stress, increased hyperlocomotion and memory impairment	Golubeva et al. (2015)
Social disruption stress	Mice, C57BL/6	Male/6–8 weeks old	–	Disruption epithelial and mucus layer (colon)	Increase in relative abundance of *Muribaculaceae, Enterorhabdus, Marvinbryantia*, *Candidatus Arthromitus* and decreased *Lachnospiraceae.*	Systemic bacterial translocation	Allen et al. (2022)
Social stress	Hamster	Male/Adult	–	–	Reduced Lactobacillus, increased *Clostridium*	–	Partrick et al. (2018)
Social disruption stress	Mice/CD-1	Male/6–8 weeks old	–	–	Decreased the relative abundance of *Bacteroides*, increased the relative abundance of *Clostridium*	Increased circulating levels of IL-6 and MCP-1	Bailey et al. (2011)
Chronic psychosocial stress	Mice, C57BL/6	Male/8 weeks old	–	–	Reduced richness and diversity of the gut microbial community	Deficits in social exploration, dendritic cell activation, and transiently elevated levels of IL-10+ T regulatory cells and IL-6 in serum	Bharwani et al. (2016)
Chronic psychosocial stress	Mice, C57BL/6	Male/8–10 weeks old	–	–	Increased *Firmicutes to Bacteroidetes* ratio	Increased T helper cells in mesenteric lymph nodes	Werbner et al. (2019)
Psychosocial stress	Mice/C57BL6	Male/8 weeks old	Antibiotics	–	–	Psychosocial stress did not induced anhedonia in ABX-treated mice. Psychosocial stress increased IL-6 levels in plasma	Wang et al. (2020)
**Clinical evidence**							
Stress perception	Healthy volunteers	–	–	–	Decreased *Bacteroidetes* and *Firmicutes*	–	Almand et al. (2022)
Stress perception	Healthy volunteers	23 years	–	Increased gastrointestinal symptoms	Reductions in total acid lactic-producing bacteria	–	Knowles et al. (2008)
Psychological and physical stress	Soldiers	–	–	Increased gastrointestinal symptoms and intestinal permeability	–	Increased IL-6 and TNF-α in serum	Li et al. (2019)
Perceived stress	Healthy volunteers	Male and female/18–45 years old	*L. acidophilus* and *B. longum*	Beneficial effects on gastrointestinal symptoms experienced by chronic stress	–	–	Diop et al. (2008)
Non stressed	Healthy volunteers	Male and female (18–60 years old)	GOS	–	–	Salivary cortisol awakening response was significantly lower after B-GOS intake compared with placebo	Schmidt et al. (2015)
Perceived stress	Healthy volunteers	Male and female (18–59 years old)	Psychobiotic diet	–	Psychobiotic diet altered microbiota composition	Psychobiotic diet reduced perceived stress	Berding et al. (2023)

Microbiota and immune system alterations have been described in rodents stressed during early life, suggesting that corticosterone might influence specific bacterial populations contributing to stress-related conditions. For instance, maternal separation was found to induce short-term transient reductions of *Lactobacilli* in rhesus monkey (Bailey and Coe, 1999). Long-term effects on the microbiome have also been reported in adult rats exposed to maternal separation early in life, when compared to rats not subjected to separation (O'Mahony et al., 2009). Rats exposed to early-life stress, with higher *Akkermansia* spp. and low *Rikenella* spp. abundance in adulthood, exhibited lower baseline levels of corticosterone (Pusceddu et al., 2015). Moreover, rats with reduced cyanobacteria and increased *Oscillobacter* spp. showed a diminished corticosterone response to a physical stressor (Golubeva et al., 2015).

Furthermore, social stressors have been shown to induce long-lasting disturbances in the gut microbiota (Allen et al., 2022; Bastiaanssen et al., 2021; Partrick et al., 2018). Repeated social stress increased the levels of proinflammatory cytokines in plasma, which correlated with three bacterial genera (i.e., *Coproccocus*, *Pseudobutyrivibrio* and *Dorea*). This suggests that social stress may influence gut microbiota in a cytokine-dependent manner (Bailey et al., 2011). Complex structural changes in the gut microbiota have also been described after chronic social stressors and have been associated with behavioral deficits and with immunoregulatory changes (Bharwani et al., 2016; Werbner et al., 2019). In this context, mice susceptible to repeated social stress exhibited specific alterations in gut bacteria, which were correlated with social avoidance behaviors and linked to changes in prefrontal cortex interleukin expression (Szyszkowicz et al., 2017).

Despite the extensive preclinical evidence, less is known from a clinical perspective. Previous studies established that chronic stress is associated with altered gut microbiota composition in humans (Almand et al., 2022; Bastiaanssen et al., 2021; Knowles et al., 2008). Increases in perceived stress seem to elucidate a decrease in potentially beneficial *Bacteroides*, with a loss in *Firmicutes* phyla in healthy participants (Almand et al., 2022). In this line, during periods of heightened stress, such as those experience by undergraduate students, perceived stress was associated with reduced gut bacterial levels, specifically in lactic acid-producing bacteria, which also noted an increased susceptibility to gastrointestinal disorders (Knowles et al., 2008). Prolonged stress due to combat training also increased the severity of gastrointestinal symptoms, pro-inflammatory immune activation (increased IL-6 and TNF-α in serum) and intestinal permeability (X. Li et al., 2014). Chronic stress has been linked to either constipation or diarrhea (Konturek et al., 2011), along with gastrointestinal complications such as inflammatory bowel disease, characterized by hypersensitivity, immune system up-regulation, alterations in behavior, and changes in the gut microbiota (Clarke et al., 2009). These findings highlight the role of prolonged stress in disrupting gut function, leading to local and systemic inflammation, and influencing microbial diversity in humans.

### 3.2 The influence of gut microbes on how the host respond to stress

The fact that the brain control gut functions is widely accepted, but recent focus has shifted towards understanding how microbes can influence the brain. Various experimental models and conditions have been employed to investigate the impact of microbiota on stress-related behaviors. These encompass the use of antibiotic cocktails, interventions with pre- and probiotics, fecal material transplantation and the use of germ-free animals (Table 1).

Early research demonstrated that animals devoid from their gut microbiota from birth (germ-free; GF), exhibited an exaggerated HPA axis activity, with elevated levels of adrenocorticotropic hormone and corticosterone when subjected to acute stress. However, these heightened stress responses returned to normal levels after GF animals were colonized with the gut microbiota obtained from control animals (Clarke et al., 2013; Sudo, 2012; Sudo et al., 2004). Furthermore, alterations in the innate immune system in response to acute stress has been reported in GF mice, highlighting the influence of the gut microbiota in priming and recovery of the immune system and stress response (van de Wouw et al., 2020). GF rodents also exhibited reduced anxiety-like behaviors in stressful novel environments, such as elevated plus maze, light/dark box and open field tests (Clarke et al., 2013; Neufeld et al., 2011). Moreover, stress-sensitive GF rats exhibited exacerbated neuroendocrine responses, which included elevated serum corticosterone levels, increased expression of corticotropin-releasing hormone gene in the brain, and changes in dopaminergic turnover in brain regions responsible for regulating stress and anxiety (Crumeyrolle-Arias et al., 2014). Another key study using GF mice demonstrated that short-term recolonization, as well as colonization with *Blautia coccoides* and *Bifidobacterium infantis,* reduced anxiety levels compared to GF mice and increased monoamine turnover in different brain regions (Nishino et al., 2013). Moreover, colonizing GF mice with indole-producing bacteria, increased vulnerability to stress, highlighting the role of bacterial metabolites in stress sensitivity (Mir et al., 2020).

An alternative method for exploring the impact of the gut microbiota on stress-related molecular and behavioral outcomes involves the use of antibiotics to reduce the gut bacterial load. However, there is a notable lack of preclinical and clinical research examining the impact of antibiotic administration on stress response alterations. A recent study has reported that microbiota depletion with a cocktail of antibiotics contributed to resilience to anhedonia in mice exposed to a repeated social stressor, providing further evidence of the critical role of gut microbes in stress-related disorders (Wang et al., 2020). Fecal material transplants are also another method widely used to explore the causative role of the gut microbiota. Emerging evidence showed that rats exposed to chronic mild stress or that received a fecal material transplant from stressed animals induced behavioral changes and increased neuroinflammatory markers in the hippocampus. Moreover, in the same study, fecal transplant from healthy donors alleviated stress-induced alterations on brain and behavior, but only when the vagus nerve was intact (Marcondes Ávila et al., 2020), suggesting that the gut microbiota may regulate stress via the vagus nerve or microbial-derived metabolites acting on this nerve (Fulling et al., 2019; Pradhananga et al., 2020). A recent study also demonstrated that perturbations of the gut microbiota induced by chronic stress activate the vagus nerve inducing brainstem neurotransmission deficits and neuroinflammation (Siopi et al., 2023), putting the vagus nerve at the interface of the microbiota-immune-brain axis (Bonaz, 2022; Bonaz et al., 2021).

Specific strains of probiotic are emerging as potential therapeutics for stress-related disorders. In a maternal separation model, *Bifidobacterium infantis* treatment restored behavioral deficits and attenuated immune changes induce by this early-life stress model, without restoring the corticosterone response (Desbonnet et al., 2010). However, a similar study found that the administration of *Lactobacillus* species partially reduced the corticosterone levels in a maternal separation model (Gareau et al., 2007). Another work reported that maternal probiotic administration of *Bifidobacterium animalis* and *Propionibacterium jensenii* induced activation of neonatal stress pathway, which persisted into adulthood. However, it appeared to protect the rats against immune dysfunction and, to some extent, disturbances in gut microbiota in adulthood induced by maternal separation and adult restraint stress (Barouei et al., 2012). Beneficial effect on stress-induced elevation in corticosterone have been observed after the administration of *Lactobacillus rhamnosus* (JB-1), an effect that disappeared in vagotomized animals, suggesting the impact of this specific bacteria on the central nervous system at a physiological level (Bravo et al., 2011). Another study demonstrated prevention of gut leakiness and also attenuation of the HPA response to an acute stressor after the administration of *Lactobacillus farciminis* (Ait-Belgnaoui et al., 2012). The same authors also reported that a pretreatment with *Lactobacillus helveticus* R0052 and *Bifidobacterium longum* R0175 attenuated HPA axis response to chronic stress, reducing the plasmatic levels of corticosterone, adrenaline and noradrenaline in stressed mice (Ait-Belgnaoui et al., 2014). Prebiotics have also shown effectiveness in mitigating the effects of stress. Specially, the administration of FOS (fructooligosacharides) and GOS (galactooligosacharide) was able to reduce corticosterone levels following acute exposure to the forced swim test, indicating an impact on stress and anxiety in socially stressed mice (Burokas et al., 2017). In line with this, supplementation with a combination of microbial metabolites (i.e., butyrate, acetate and propionate) alleviated enduring alterations induced by repeated psychosocial stress, suggesting the potential of microbial targeted therapies for stress-related disorders (van de Wouw et al., 2018).

In clinical studies, the administration of *Lactobacillus acidophilus* and *Bifidobacterium longum* provided beneficial effects on gastrointestinal symptoms experienced by individuals affected by chronic stress (Diop et al., 2008). In this regard, a combination of two bacterial strains *Lactobacillus helveticus* R0052 and *Bifidobacterium longum* R0175 display anxiolytic-like activity in rats and a reduction in stress, anxiety and depression in healthy human volunteers, indicating beneficial psychological effects (Messaoudi et al., 2011). The intake of GOS daily for three weeks notably reduced salivary cortisol awakening response in healthy participants compared to placebo (Schmidt et al., 2015), demonstrating central effects of prebiotics in humans. Recently, dietary approaches have reported beneficial effects on improving stress perception in healthy volunteers. These results highlight the use of gut microbiota-targeted therapies to positively modulate gut-brain communication, potentially reducing stress and stress-associated disorders (Berding et al., 2023).

## 4 Social behavior and the microbiome

### 4.1 Sociability can affect gut microbial composition

For the most part, it is thought that mammals lack gut microbiota prior to birth, resulting that each new generation must undergo a process of assembling their gut microbiota (Cabré et al., 2022; Kennedy et al., 2023). This phase of establishing the gut microbiome involves both vertical transmission of maternal microbiota and horizontal transmission from other members of the group or environmental microorganisms (Pinacho-Guendulain et al., 2022). The colonization is crucial in safeguarding the newborn against pathogens, especially when the immune system has not yet fully developed (Gensollen et al., 2016).

In social animals, social interactions can influence the horizontal transmission of gut microbes, leading to changes in the taxonomic and genetic composition of the microbiome (Table 2). This social transmission may vary among species (Sherwin et al., 2019), enhancing the transmission of beneficial microbes, favoring stability and resilience of the gut microbiome, and helping protect the host from potential pathogens (Johnson et al., 2022). In invertebrates, behaviors like coprophagia and trophallaxis play a significant role in the transmission of microbes (Engel and Moran, 2013; Koch and Schmid-Hempel, 2011; Martinson et al., 2012). Similarly, in rodents, coprophagy serves as a way of obtaining food and as a mechanism for transmitting microbiota, thereby promoting social interactions (Archie and Tung, 2015). In humans and primates, the social transfer of microbiota occurs among individuals sharing specifics environments or lifestyles, such as having common microbial sources and dietary habits (Sarkar et al., 2020; Valles-Colomer et al., 2023). Primates within the same social groups, as well as humans residing in the same household, tend to exhibit microbial communities that are more similar than those found in individuals within their broader population (Degnan et al., 2012; Lax et al., 2014; Song et al., 2013; Tung et al., 2015). Similar gut microbes has been also found in married couples who rate their relationship as specially close (Dill-McFarland et al., 2019). These changes may have important consequences for the functions that microbes perform for their hosts.

**Table 2:** Key studies evaluating social behaviors and the microbiota-gut-brain axis.

Species, strain	Sex/age	Microbiota modulation	Gut	Taxonomy	Function	Work
**Preclinical evidence**						
Rhesus macaques	–	–	–	*Faecalibacterium* correlated with sociability and *Streptococcus* abundant in less social macaques	*Faecalibacterium* beneficial effects on health (anti-inflammatory properties). *Streptococcus* pathogenic specie	Johnson et al. (2022)
Mice	Male/Adult	Germ-free	–	–	Reduced social interaction	Desbonnet et al. (2014)
Mice/Swiss-Webster	Male/3 month old	Germ-free	–	–	Increased social interaction	Arentsen et al. (2015)
Mice/Swiss-Webster	Male/9–10 weeks old	Germ-free	–	–	GF showed dendritic hypertrophy in aspiny interneurons and pyramidal neurons in amygdala. GF showed atrophy in dentate granule cells of the hippocampus	Luczynski et al., 2016
Mice/NIH Swiss	Male/PND 55-80	–	–	–	Decreased social transmission of food transmission	Desbonnet et al. (2015)
Mice/C57BL6	Male/8 weeks old (offspring)	Antibiotic to dams	–	–	Maternal ABX exposure altered sociability on offspring	O'Connor et al. (2021)
Mice/C57BL6	Male/6 weeks old	Antibiotic	Decreased pro-inflammatory interleukins (ileum)	–	ABX exposed mice increased social reward	García-Cabrerizo et al. (2023)
Mice/C57BL6	Male/9–10 weeks old and 18 months old	Antibiotic	–	–	ABX exposure restores the age-dependent social cognitive defects and modulates T-Helper cell numbers in the choroid plexus	Cruz-Pereira et al. (2023)
Mice/C57BL6	Male/Adult	p-cresol		p-cresol increased *Duncaniella dubosii*, *Barnesiella* sp., *Muribaculaceae bacterium*, *Anaerobium* sp., and *Turicimonas muris*	p-cresol decreased sociability	Bermúdez Martín et al., 2021
Mice/C57BL6	Male/7–12 weeks old	*L. reuteri*	–	–	*L. reuteri* treatment restores oxytocin levels, VTA plasticity and social behaviors	Buffington et al. (2016)
Mice/C57BL6 wild type, Shank3b −/− and BTBR	Male/Adult	Germ-free and *L. reuteri*	–	–	*L. reuteri* rescues social deficits in several ASD mouse models and GF mice	Sgritta et al. (2019)
Mice/C57BL6	Male/Adult	*E. faecalis*	–	–	*E. faecalis* restores social behavior and reduced corticosterone levels after social stress	Wu et al. (2021)
Mice/C57BL6	Male/7 weeks old	FOS and GOS	–	–	GOS and FOS improved sociability in stressed mice	Burokas et al. (2017)
**Clinical evidence**						
Human	Male/Female	–	–	*-*	Gram-negative bacteria displayed enhanced gut maternal and household transmissibility	Valles-Colomer et al. (2023)
Human	Male/Female	–	–	–	Cohabiting increased alpha diversity. *Lachnospiraceae* and *Ruminococcaceae* commonly families shared	Dill-McFarland et al. (2019)
Children with autism	Male/Female (4–11 years)	B-GOS	B-GOS improved gastrointestinal symptoms	B-GOS increased *Bifidobacterium* spp., *Ruminococcus* spp., *Lachnospiraceae* family, *Eubacterium dolchum*, TM7–3 family and *Mogibacteriaceae.*	B-GOS improved anti-social behaviors in autistic children	Grimaldi et al. (2018)
Healthy children	Male/female (7–16 years)	Omega-3	–	–	Omega-3 reduced aggressive and antisocial behaviors	Raine et al. (2019)

In bumblebees, microbiota transfer from nest mates to emerging adult is essential for defense against parasites that affects the fertility of the queen, indicating a clear positive effect (Koch and Schmid-Hempel, 2011). Although parasites have long been known to manipulate host behaviors to improve transmission, bacteria could also benefit from behavioral manipulation. Some research suggests that gut microbiota has evolved to manipulate their hosts, while hosts genes have evolved to counteract microbial manipulation that conflicts with their own interests (Alcock et al., 2014; Kurokawa et al., 2007; Qin et al., 2010). Changes in the composition of the bacterial community can disrupt the initially beneficial interaction, contributing to the development of various disorders, such as eating disorders (DeGruttola et al., 2016; Glenny et al., 2017). For example, *Proteus mirabilis* is a bacterium present in the salivary glands of flies that produced volatile compounds to attract other flies to new foods favoring their expansion to other members and environments (Ma et al., 2012). Social interactions can also favor the transmission of pathogens within the group, promoting their expansion and taking advantage to colonize new individuals at the expense of the host. Nevertheless, this pathogenic transmission could have a positive effect on the group, achieving a collective group defense or social immunity against future infections mitigating the spread (Schmid-Hempel, 2017). On the other hand, certain gut bacteria such as *Klebsiella pneumonia* and *Proteus mirabilis*, are associated with a high incidence of colitis. Interestingly, the effects of social transmission differ when wild-type mouse pups are nursed by susceptible mothers, they become susceptible to colitis, but when susceptible pups are nursed by wild-type mothers, both the microbes and colitis susceptibility disappear (Garrett et al., 2010).

### 4.2 Social microbes helping us to shape our social behaviors

Sociability is a fundamental behavior that significantly impact behavioral outcomes, including learning, cooperation, protection and mating. It is intriguing to note that social behavior appears to be under the influence by the gut microbiota across the animal kingdom (Sherwin et al., 2019) (see Table 2).

This phenomenon has been observed in GF mice, which exhibited reduced interaction time with unfamiliar conspecific when compared to conventionally colonized mice (Desbonnet et al., 2014). Conventionally colonized mice, on the other hand, display a preference for spending more time a novel unfamiliar mouse compared to a familiar one. In contrast, GF mice fail to differentiate between novel and familiar mice, indicating cognitive deficits in recognizing social novelty. Reconstituting the gut microbiota in GF mice improved their interaction with unfamiliar mice compared to inanimate objects but did not ameliorate their ability to discern social novelty (Desbonnet et al., 2014). However, other investigations using GF mice reported contradictory results, such as enhancement in sociability and social cognition (Arentsen et al., 2015). Despite the varied results, the observations suggest that manipulating the gut microbiota might alter certain social aspect in mice. In this line, alterations in the amygdala, a region implicated in social behaviors, have been described in rodents subjected to microbial manipulations. Morphological analysis in GF mice revealed an extensive neuronal hypertrophy and dendritic arborization of aspiny interneurons and pyramidal neurons in the basolateral amygdala leading to a volumetric increase of various amygdala nuclei (Luczynski et al., 2016b). Furthermore, transcriptional factors associated with stress, anxiety and neurotrophins exhibited changes in the amygdala of GF mice, and these changes subsequently reversed upon microbial colonization (Cowan et al., 2018).

Antibiotics have been a useful tool to unravel the potential role of the gut microbiota in social behaviors. Several works have documented that antibiotics administration, resulting in marked reduction in gut microbial diversity is associated with deficits in social behaviors. Pregnant rat dams exposed to a non-absorbable antibiotic induced a reduction in social interactions both in male and female offspring, indicating that maternal microbiota provokes alteration in the offspring behaviors related with sociability (Degroote et al., 2016). In line with this, the administration of antibiotics from weaning reduced the expression of oxytocin and vasopressin in the adult brain, indicating that the gut microbiota alters key neuromodulators associated with social behaviors (Desbonnet et al., 2015; O'Connor et al., 2021). The effects of antibiotics on reducing social cohesion has also been observed in zebrafish (Berer et al., 2011), demonstrating that these effects on social behaviors are not limited to mice (Nagpal and Cryan, 2021). Recent studies have pointed out the depleting the gut microbiota with cocktails of non-absorbable antibiotics could positively modulate social behaviors. In particular, the administration of antibiotics alter the effect of social stimulus on reward behavior in mice, suggesting that depleting the gut microbiota favor social reward and reduces the levels of proinflammatory interleukins in the ileum (García-Cabrerizo et al., 2023). Similarly, it has been reported that gut microbiota depletion reverse age-dependent social deficits in mice and also reduced the accumulation of T cells in the choroid plexus induced by ageing (Cruz-Pereira et al., 2023). The mechanism responsible for microbiota-induced regulation of social behavior remain unknown and are likely to involve a complex interplay of diverse biological pathways. Fecal material transplants have revealed that microbial metabolites such as p-cresol, metabolite linked to autism spectrum disorders, induced social deficits in a microbiome-dependent mechanism (Bermudez-Martin et al., 2021). Alterations in gut microbiota observed in mice susceptible to traumatic stress increased the production of p-cresol, affecting the dopaminergic system and suggesting that microbial metabolites may influence key brain areas involved in stress and social behaviors (Laudani et al., 2023). A recent study provided new evidence indicating that the microbiota of individuals with social anxiety disorder may induce heightened social fear, impaired peripheral immune response, and altered neuronal oxytocin levels in the bed nucleus of the stria terminalis in mice who underwent a fecal material transplant (Ritz et al., 2024a). This suggests that the microbiota could play a causal role in heightened social fear responses in the disorder, indicating that targeting the microbiota-gut-brain axis could be a promising approach for developing new therapeutics to alleviate symptoms in social disorders.

Based on the evidence that gut microbes can influence social behaviors, it is logical to conclude that altering the gut microbiota with specifics pre- or probiotic supplementation or dietary interventions could likewise impact sociability. For example, mice born from mother exposed to high-fat diet exhibited decreased sociability, reduced oxytocin production, synaptic deficiency in the ventral tegmental area and a reduction in *Lactobacillus* species. However, the administration of *Lactobacillus reuteri* restored oxytocin levels, ventral tegmental area plasticity and social behaviors in these mice (Buffington et al., 2016). The positive effect of *Lactobacillus reuteri* on oxytocin and social behaviors has been observed in different animals models of autism spectrum disorder, a neurodevelopmental disorder characterized by alterations in social behavior (Buffington et al., 2016; Sgritta et al., 2019; Tabouy et al., 2018). Furthermore, the prosocial effects triggered by *Lactobacillus reuteri* relied on the integrity of the vagus nerve, as the rescue of social deficits seen in the autism spectrum disorder genetic mouse model was not evident in vagotomized mice (Sgritta et al., 2019). Using a well-characterized mouse model of liver inflammation (i.e., bile duct ligation), where mice exhibited decreased social behaviors, the administration of a probiotic cocktail (VSL#3, comprising different *Lactobacilli*, *Bifidobacteria*, and *Streptococcus*) not only ameliorated the social behavior reduction but also lowered the levels of circulating TNF-α (D'Mello et al., 2015). A recent study suggested that specific gut bacteria, such as *Enterococcus faecalis,* can restrain the activation of the HPA axis, affecting social behaviors through neuronal circuits that mediate stress responses in the brain (Wu et al., 2021). Prebiotics such as GOS and FOS improved sociability in stressed mice (Burokas et al., 2017). The administration of B-GOS and exclusion diets improved gastrointestinal and social behaviors in children with autism spectrum disorders, indicating that multiple interventions resulted in changes in the gut microbiota and metabolism being more relevant in the improvement of psychological traits (Grimaldi et al., 2018). Dietary interventions also affect the gut microbiota and social behaviors. A deficiency in omega-3 fatty acids impairs social behaviors in rat, while supplementing this compound can prevent social deficits and reduce HPA axis activation, in addiction to modifying gut microbiota composition and mitigating inflammation (Robertson et al., 2017). Improvements in social communication in autistic children, as well as reductions in aggressive and antisocial behaviors in children, have been reported after the administration of omega-3 (Doaei et al., 2021; Raine et al., 2019).

## 5 Addiction and the microbiome

### 5.1 Drugs of abuse as a modulator of the gut microbiota

Recent evidence supports the concept that substances of abuse have an impact on the gut microbiota and these changes might contribute in the development of addiction disorders (Acharya et al., 2017; Kirpich et al., 2008; Leclercq et al., 2014; Mutlu et al., 2009; Ning et al., 2017; Scorza et al., 2019; Volpe et al., 2014; Wang et al., 2012) (see Table 3). Besides the direct impact of drug of abuse on the gut microbiota, addiction also encompasses numerous coexisting conditions that have previously demonstrated associations with the microbiota-gut-brain axis. These include stress, anxiety, depression and inflammatory processes (Cryan et al., 2019).

**Table 3:** Key studies evaluating drugs of abuse and the microbiota-gut-brain axis.

Species, strain	Sex/age	Drug	Microbiota modulation	Gut	Taxonomy	Function	Work
**Preclinical evidence**							
Mice/C57BL6	Male/Adult	Alcohol	–	–	Alcohol reduced *Firmicutes* and increased in *Verrucomicrobia* and *Bacteroides*	Alcohol Bacterial increased translocation to mesenteric lymph nodes and blood	Yan et al. (2011)
Mice/C57BL6	Male/8–10 weeks old	Alcohol	*L. rhamnosus GG*	–	Alcohol decrease the relative abundance of *Bacteroides* and *Fermicutes*, as well as increased the abundance of *Proteobacteria* and *Actinobacteria*	Alcohol intake induced intestinal barrier dysfunction and liver disease. *L. rhamnosus* GG ameliorated alcohol-induced endotexemia and hepatic inflammation.	Bull-Otterson et al. (2013)
Mice/C57BL6	Male/Adult	Alcohol	–	–	Alcohol increase in *Alistipes* and reduced in *Clostridium IV* and *XIVb*, *Dorea* and *Coprococcus*	Bacterial changes found align with previous findings associated to inflammation	Peterson et al. (2017)
Rat/Wistar	Male/Adult	Alcohol	–	–	Increased *Firmicutes* and decreased *Actinobacteria* in alcohol Vulnerable rats	Significant correlations were observed between microbiome and dopamine receptors (increased dopamine 1 and decreased dopamine 2 receptors)	Jadhav et al. (2018)
Rat/Wistar	Male/female	Cocaine	–	–	Decreased in alpha diversity	–	Scorza et al. (2019)
Mice/C57BL6	Male/Adult	Cocaine	–	–	Increased *Citrobacter rodentium*	Cocaine raises gut norepinephrine levels facilitating Proteobacteria colonization. Glycine depletion alters cocaine-induced neuroplasticity and drug responses.	Cuesta et al. (2022)
Rat/Sprague-Dawley	Male/Adult	Methamphetamine	–	–	Increased *Bacillaceae* and *Ruminococcaceae* and decreased *Acidaminococcaceae*	Methamphetamine decreased the levels of propionate	Ning et al. (2017)
Mice/C57BL6	Male/Adult	Methamphetamine	–	Reduction of tight junction proteins and increased permeability (colon)	Reduced the abundance of *Muribaculaceae*, *Prevotellaceae*, and *Lactobacillaceae and increased Proteobacteria*	Increased pro-inflammatory markers (TNF-α and IL-1β) in serum	Wang et al. (2022)
Mice/C57BL6	Male	Morphine	–	Increased gut permeability and inflammation in small intestine	–	Morphine induced bacterial translocation in liver and mesenteric lymph node	Meng et al. (2013)
Mice/C57BL6	Male	Morphine	–	–	Decreased *Bacteroidetes* and increased *Firmicutes*	Morphine-induced reversal of endotoxin tolerance	Banerjee et al. (2013)
Mice/C57BL6	Male	Morphine	–	–	Increased *Enterococcus faecalis*	–	Wang et al. (2018)
Rat/Sprague-Dawley	Male/Adult	Fentanyl	Antibiotics	–	Fentanyl did not alter the microbiome	Antibiotics increased fentanyl seeking behavior	Hofford et al. (2022)
Rat/Sprague-Dawley	Male/Female/Adult	Fentanyl	–	–	Fentanyl changes gut bacteria alpha diversity	Gut bacteria alpha diversity predicts progressive ratio responding in female rats	Ren and Lotfipour (2022)
Rat/Wistar	Male/Adult	Alcohol	Antibiotics and L. *rhamnosus GG*	ABX reduced intestinal inflammation	–	Antibiotics inhibit voluntary ethanol intake. *L. rhamnosus* reduced voluntary ethanol intake.	Ezquer et al. (2021)
Mice/C57BL6	Male/6 weeks old	Cocaine	Antibiotics	Decreased pro-inflammatory interleukins (ileum)	–	Antibiotics exposed mice decreased cocaine reward	García-Cabrerizo et al. (2023)
Mice/C57BL6	Male/6–8 weeks old	Cocaine	Antibiotics	ABX alters microglia morphology	–	Antibiotics exposed mice decreased cocaine reward	Lee et al. (2018)
Mice/C57BL6	Male/7–9 weeks old	Morphine	Antibiotics	–	Morphine did not alter alpha diversity	Antibiotics exposed mice decreased morphine reward and locomotor sensitization	Hofford et al. (2021)
Mice/C57BL6	Male/3 weeks old	Fecal material transplant from human alcoholics	Fecal material transplant	–	–	Fecal material transplant impaired sociability and reproduce metabolic alterations associated with alcohol dependence	Leclercq et al. (2020)
Mice/C57BL6	Male/Adult	Fecal material transplant from methamphetamine users	Fecal material transplant	–	–	Methamphetamine altered microbiota promoted anxiety and depression-like behaviors in recipient mice	Yang et al. (2022)
Mice/C57BL6	Male/Adult	Morphine	Germ-free and VSL#3	Morphine-induced dysbiosis, disrupted gut epithelial barrier and promoted systemic bacterial translocation.	–	Morphine tolerance is associated with selective depletion in *Bifidobacteria* and *Lactobacillaeae*. Germ-free mice showed an attenuation on analgesic tolerance. Probiotic VSL#3 attenuates morphine tolerance and prevents morphine-induced gut microbiota alterations	Zhang et al. (2019)
**Clinical evidence**							
Individual with alcohol addiction	Male/Female	Alcohol	–	Increased leaky gut	Decrease in *Ruminococcaceae* and increased Lachnospiraceae	Gut microbiota alterations could alter the gut-barrier function and influence behavior in alcohol dependence.	Leclercq et al. (2014)
Healthy subjects	Male/Female	Alcohol	–	–	High binge drinking reduced *Alistipes* and increased *Veillonella dispar*.	Associations were found for several microbiome species with emotional processing and impulsivity. Craving showed a strong link with alterations in microbiome composition and neuroactive potential over time. number of binge drinking episodes correlated with higher responsiveness of stimulated cytokines (IL-6 and IL-8)	Carbia et al. (2023)
Humans	Male/Female	Cocaine	–	–	Increased *Bacteroidetes*	–	Volpe et al. (2014)
Human users	Male	Methamphetamine	–	–	*Ruminococcaceae, Rikenellaceae*, and *Enterobacteriaceae* were significantly higher in methamphetamine users. Lower levels of *Bacteroidaceae* and *Alcaligenaceae* in methamphetamine users	–	Yang et al. (2022)

One of the drugs of abuse more extensively studied in the context of the gut is alcohol. Chronic alcohol intake has been associated with marked alterations in gut microbial diversity, increased in leaky gut and hepatic inflammation. Mice intragastrically fed with alcohol exhibited alcohol-induced liver disease, which correlated with reductions in Firmicutes and increases in Verrucomicrobia and Bacteroides (Yan et al., 2011). Furthermore, mice chronically fed with alcohol decrease the relative abundance of Bacteroides and Fermicutes, as well as increased the abundance of Proteobacteria and Actinobacteria in feaces (Bull-Otterson et al., 2013). Other models to induce alcohol dependence, such as chronic intermittent vaporized alcohol exposure, also alters the gut microbiota in mice, reporting an increase in the genus *Alistipes* and reduction in the genera *Clostridium IV* and *XIVb*, *Dorea* and *Coprococcus* (Peterson et al., 2017). Alterations in the caecum gut microbiota induced by the alcohol intake has been associated with an increase in impulsive behaviors and alterations in dopaminergic receptors in the striatum, suggesting that changes in the microbiota could affect vulnerability to alcohol use disorders (Jadhav et al., 2018). Furthermore, intermittent ethanol exposure during adolescence induced immediate and long lasting microbial and neurotransmitter alterations in the gut, that may contribute to psychological symptoms associated with alcohol use disorders (Vetreno et al., 2021). In humans, patients with alcohol-induced liver injury had a decrease in *Lactobacilli*, *Enterococci* and *Bifidobacteria* (Kirpich et al., 2008). Furthermore, changes in gut permeability and microbiota composition (decrease in *Ruminococcaceae* family) has been associated with alcohol craving (Leclercq et al., 2014). In this context, alterations in the gut microbiome have been observed in young binge drinkers, identifying potential early biomarkers of craving and highlighting the relevance of new gut-derived interventions to improve early alcohol-related alterations (Carbia et al., 2023).

Other drugs such as stimulants and opiates are highly addictive substances and also has an impact in the gut microbiota, although these alterations have not been studied in depth. A few studies have shown that cocaine can induce alterations in the gut microbiota. For instance, rats chronically exposed to cocaine, as well as to the adulterants caffeine and phenacetin, leads to alterations in the gut microbiota (Scorza et al., 2019). A recent study conducted in mice revealed that repeated cocaine can lead to changes in the levels of host catecholamines, which can be sense by γ-Proteobacteria pathogens. These pathogens subsequently deplete host metabolites, influencing cocaine-induced plasticity and addiction-like behaviors (Cuesta et al., 2022). In humans, stool sample from cocaine users presented higher abundance of Bacteroidetes when compared with non-users (Volpe et al., 2014). Other stimulants such as methamphetamine reported increases in the relative abundance of Proteobacteria and Fusobacteria in rats (Ning et al., 2017). Mice repeatedly treated with methamphetamine, induced intestinal injury and might be attributed to gut microbiota dysbiosis and an overproduction of lipopolysaccharide (Wang et al., 2022). Methamphetamine users also exhibit alterations in their microbial community that might be associated with the gastrointestinal impairments observed in the users (Yang et al., 2022). Opioids are drugs that have shown significant alterations in the gut. Morphine has been shown to impair the integrity of the gut epithelial barrier by causing damage to tight junction proteins through the activation of Toll-like receptors in mice (Meng et al., 2013). This disruption could potentially lead to conditions like sepsis and immune system dysregulations (Banerjee et al., 2013). Brief exposure to morphine rapidly triggered changes in the gut microbiota, including an increase in communities linked to pathogenic activity and a decreased in those associated with stress tolerance (Wang et al., 2018). Recent reports indicate that changes in gut microbiota composition in response to intravenous fentanyl exposure are influenced by both sex and dosage in rats (Hofford et al., 2022; Ren and Lotfipour, 2022), suggesting that the effects of opioids on the microbiome composition may vary on factors such as type of opioid, dosage, frequency and rout of administration. These findings suggest that exposure to drugs of abuse induces potential changes in the gut microbiota, which could correlate with altered stress and reward processing, potentially influencing the pathogenesis of substance use disorders through complex microbial and neurological pathways.

### 5.2 Gut microbes and substance use disorders

Evaluating how alterations in the gut microbiota can potentially impact behaviors linked to drug abuse has been of significant importance (see Table 3). Recently, it has been reported morphological alterations in medium spiny interneurons in the nucleus accumbens of germ-free mice (Garcia-Cabrerizo et al., 2024). These morphological alterations observed in this key brain region of the brain reward pathway may have implications for behavioral and physiological outcomes relevant to stress, depression and drug addiction. Indeed, marked baseline gene dysregulations have been also reported in the nucleus accumbens of germ-free mice, providing evidence of the potential developmental effects of the gut microbiome on brain signaling and the plasticity response to external stimuli such as drugs of abuse (Sens et al., 2023). However, these findings should be interpreted with caution, as there are potential secondary influences of GF mice which might not be primarily form their germ-free status. This could encompass broader alterations in development (i.e., affecting both the brain and body) over multiple generations within a GF colony which could be attributed to epigenetic mechanisms among other variables (Luczynski et al., 2016a).

Depleting the gut microbiota with antibiotics to induce negative modulations has generated valuable insight into the potential involvement of gut microbes in drug addiction. However, the outcomes have been diverse. For instance, studies have demonstrated that depleting the gut microbiota with antibiotics can lead to decreased voluntary ethanol consumption in rats bred for high ethanol intake, coupled with a reduction in pro-inflammatory markers in the colon (Ezquer et al., 2021). It is also crucial to emphasize how modifications in the gut microbiota can bring shifts in drug reward. Initially, it was reported that administering antibiotics orally increased conditioning and sensitization at sub-threshold cocaine doses (Kiraly et al., 2016). However, recent research has revealed that depleting gut microbes results in reduced cocaine conditioning (García-Cabrerizo et al., 2023; Lee et al., 2018), as well as a decreased conditioning and sensitization to opioids like morphine (Hofford et al., 2021). Conversely, when examining self-administration patterns, research has revealed that depleting the gut microbiota can enhance behaviors related to acquiring cocaine and promote cocaine-seeking behaviors during withdrawal (Meckel et al., 2023). Similar outcomes have been observed with opioids like fentanyl, where gut microbiota depletion has amplified their reinforcing properties (Hofford et al., 2022; Ren and Lotfipour, 2022) and induced marked alterations at the proteomic level in critical brain reward regions, such as the nucleus accumbens (Hofford et al., 2022). The discrepancies observed in these distinct studies might, to some extent, result from differences in the composition of the antibiotic cocktails used and the duration of the interventions, both of which could significantly influence drug-induced reward responses. Also, it has been suggested that metabolic bioproducts from bacterial fermentation such as short-chain fatty acids, could be a crucial mediator in the microbiome-brain communication responsible for the effects on drug reward and seeking behaviors caused by microbiome depletion, positioning the gut microbiome as a potential translational target (Hofford et al., 2021, 2022; Kiraly et al., 2016; Meckel et al., 2023).

Another important work found that fecal transplantation from individuals with alcohol addiction (who exhibited impaired sociability) to mice, reproduced the metabolic and behavioral alterations associated with alcohol dependence. These changes included reduced sociability, increased depressive-like behaviors and higher stress levels (Leclercq et al., 2020). A recent study evaluating human to mouse and mouse to mouse fecal material transplantation confirmed that methamphetamine altered transplantation is sufficient to promote anxiety- and depression-like behaviors in recipient mice, supporting the idea that the gut microbiota mediate some of the methamphetamine psychiatric symptoms (Yang et al., 2022).

Preclinical studies have reported potential advantages of probiotic supplementation in the context of drug abuse. Specifically, the use of *Lactobacillus rhamnosus GG* was effective in preventing intestinal barrier dysfunctions and subsequent alcoholic liver disease (Bull-Otterson et al., 2013). Moreover, *Lactobacillus rhamnosus GG* was found to modulate dopaminergic activity, reducing the relapse to ethanol consumption, making it a promising probiotic adjuvant for intervention in the treatment of alcohol use disorders (Ezquer et al., 2022). Positive outcomes have been documented for the probiotic VSL#3, which mitigated the development of analgesic tolerance to morphine in mice. This effect was linked to a partial restoration of components of the gut microbiota and a reduction of proinflammatory cytokines (Zhang et al., 2019). However, despite these associations, there is still much that remains unknown about the underlying mechanism through which the gut microbiota and probiotics impact host physiology and behavior.

## 6 Social influence and vulnerability to drugs of abuse: gut microbes as a key player?

### 6.1 Impact of social stress during development in drug addiction

Early life and adolescence mark the most dynamic periods of change in both gut microbiota and neuronal development, whit infants acquiring their initial gut microbiota from their pregnant mothers, a process recognized as a significant contributor to neurodevelopment (Cowan et al., 2018). This initial colonization continues to evolve in adolescence, a critical period characterized by shifts in neuronal structure and function facilitating the development of behavioral and social skills (Burnett et al., 2011; Kilford et al., 2016; Lamblin et al., 2017; Spear, 2000). Epidemiological data indicates that drug experimentation during adolescence is associated with an increased risk of developing substance use disorders (Zhang et al., 2021), highlighting the vulnerability of this developmental period in substance abuse effects (García-Cabrerizo et al., 2015; García-Cabrerizo and García-Fuster, 2016, 2019; Spear, 2016). Notably, c-section delivery disrupts the vertical microbiota transmission impacting the immune as well as the dopaminergic system, resulting in alterations in neonatal brain catecholamine levels (Boksa and El-Khodor, 2003; El-Khodor and Boksa, 1997). These initial cesarean-related dopamine modifications are linked to long-term increases in D1 receptors in the nucleus accumbens and dorsal striatum during adolescence (Juárez et al., 2005) as well as elevated tyrosine hydroxylase and dopamine levels in these regions in adulthood (Boksa and El-Khodor, 2003; El-Khodor and Boksa, 1997). In this line, repeated stress in c-section rats exhibited alterations in dopamine activity in the nucleus accumbens and striatum when compared to vaginally born (Boksa and Zhang, 2008). Consequently, changes in the microbiota during early life periods and the exposure to stressors could potentially disrupt the signaling pathway between the gut and the brain, promoting neurodevelopmental alterations as well as vulnerability to drug exposure (Ganguly and Brenhouse, 2015).

Early life adversity has consistently shown to induce alterations on brain circuits, stress-responsivity and neuroinflammation (Baracz et al., 2020; Sachser et al., 2011). This increase in immune function and neuroinflammation is an adaptive response to stress to prepare the individual to a future threatening environment. However, prolonged inflammation can induce sickness behavior that is associated with social avoidance and anhedonia (Dantzer et al., 2008), demonstrating that the immune system is a powerful modulator of neuronal circuits supporting social behavior and reward. Therefore, early life and adolescent social stressors have the potential to influence the central nervous system affecting the stress response, social behaviors and increasing susceptibility to drug addiction (Buisman-Pijlman et al., 2014). Maternal separation has reported increases in ethanol consumption during adolescence (Daoura et al., 2011; Daoura and Nylander, 2011; García-Gutiérrez et al., 2016; Palm et al., 2013; Peñasco et al., 2015; Portero-Tresserra et al., 2018). However, when assessing the impact of early life social stressors on behaviors associated with psychostimulants, there have been inconsistent findings. In general, studies have revealed that animals subjected to maternal separation did not exhibit psychostimulants reward or sensitization later in adolescence (Faure et al., 2009; Martini and Valverde, 2012; Muhammad and Kolb, 2011). Nevertheless, a recent study reported that adolescence is a more vulnerable period, as compared to adulthood, to the combined impact of adolescence cocaine and early maternal deprivation, suggesting that the accumulation of stress in early life periods can anticipate the negative behavioral outcome associated with drug consumption (Bis-Humbert et al., 2020). Altogether, these results suggest the impact of social stress during early life on drug-related behaviors during adolescence may vary and could depend on the specific type of drug involved.

Adverse social experiences during adolescence such as bullying or child abuse, has several consequences in adulthood (Ehlert, 2013; Méndez Leal and Silvers, 2021; Wolitzky-Taylor et al., 2017). Social defeat model, considered a model of bullying model in mice, promote drug intake in adulthood (Burke et al., 2016, 2011; Burke and Miczek, 2014; Montagud-Romero et al., 2015; Rodríguez-Arias et al., 2017), confirming that social stressors during this developmental stage have enduring effects later in life. Adolescent exposure to social defeat stress also increased susceptibility to the rewarding effects of alcohol, and this heightened vulnerability may be linked to changes in HPA axis within mesocorticolimbic areas (Rodriguez-Arias et al., 2016). However, the impact of social stress on behavior and neurobiology varies among individuals. A clear example was observed in social defeat susceptible mice, which displayed an increase in the rewarding properties of cocaine and was associated with an increase in neuroinflammatory markers such as IL-6 (Ballestín et al., 2021). Inflammatory cytokines can modulate dopaminergic neurons of the reward pathways, altering neurotransmitter signaling and reuptake (Felger and Miller, 2012; Northcutt et al., 2015) and also are powerful modulators of social behaviors (Bluthé et al., 1994; Eisenberger et al., 2017). Anti-inflammatory drugs such as oxytocin, could prevent alcohol consumption in social defeat mice as well as decrease neuroinflammatory response in the striatum (Reguilón et al., 2021). Social isolation is used as a model of social exclusion that affects brain reward and stress system inducing depressive-like symptoms such as anhedonia in rodents (Wallace et al., 2009). Early social isolation during adolescence increase the vulnerability for drug addiction later in life, indicating that environmental social stressors can modulate brain reward, altering corticotropin-releasing hormone and the development oxytocin system in the nucleus accumbens and paraventricular nucleus (El Rawas et al., 2020; Lukkes et al., 2009; Pan et al., 2009).

Drug abuse can act as a stress inducer, prompting changes in stress mechanisms and potentially playing a significant role in increasing vulnerability to addiction when coupled with other stressors. The impact of drugs on the stress response varies depending on the class of drugs as well as the pattern of use (Wemm and Sinha, 2019). Acute effects of drugs of abuse often involve heightened cortisol/corticosterone levels and activation of the stress response (Borowsky and Kuhn, 1991; de Wit et al., 2007; Feller et al., 2014; Richardson et al., 2008). However, chronic exposure can result in intricate alterations in the HPA axis, adrenergic system, and autonomic nervous system, highlighting the complexity of drugs-induced changes (Parrott et al., 2014; Sofuoglu et al., 2001; Thayer et al., 2006). These findings suggest that stress-drug adaptations might contribute to the progression of addiction. Furthermore, when coupled with social stressors during specific susceptible periods this could serve as an additional factor amplifying vulnerability to develop addiction.

### 6.2 Social-based interventions to overcome drug addiction

A crucial consideration in the progression of drug addiction is often coupled with the social isolation and exclusion experienced by individuals struggling with addiction, making it difficult for them to reconnect with social circles (Berkman et al., 2014; Heilig et al., 2016). This self-isolation is often arising from various factors such as fear of judgement, the stigma associated with addiction, exclusion from the social environment, or the co-occurrence of other brain disorders like anxiety or depression. Feelings of social isolation and loneliness serve as aversive stimuli, contributing to perpetuation of drug use in drug addicts. This establishes a recurring cycle wherein individuals may use drugs as a coping mechanism for feelings of social isolation (Aloise-Young and Kaeppner, 2005). This highlights the importance of social stressors in the escalation of drug dependence and addressing these social deficits through interventions at a social level may prove effective in reversing drug dependence (Lemos et al., 2021). Group life is an adaptation to solve social problems, making social and non-social decision to cooperate or compete with conspecifics, balancing the costs and benefits associated with group living. This intimate contact between members also favors the transfer of microorganisms from different hosts, where individuals thought their decisions, may control the expression of certain social behaviors for selecting microbes within member of their social group (Achtman and Wagner, 2008). This raises the question of how individuals operate to select microorganisms or whether it is these microorganisms themselves that participate in their own selection by influencing the social behavior of their hosts (Pinacho-Guendulain et al., 2022). Furthermore, most preclinical investigations evaluating social interactions involve animals housed in group, resulting to a shared microbiome among them (Archie and Tung, 2015; Tung et al., 2015). However, it is important to highlight that in preclinical studies focusing on drug self-administration, animals are typically housed individually and also, they may be subjected to antibiotic exposure following intrajugular surgery, potentially impacting their microbiome and contributing to a unique microbial composition (Donovan et al., 2020; Kim et al., 2022) altering the development of drug addiction.

**Fig. 2:**
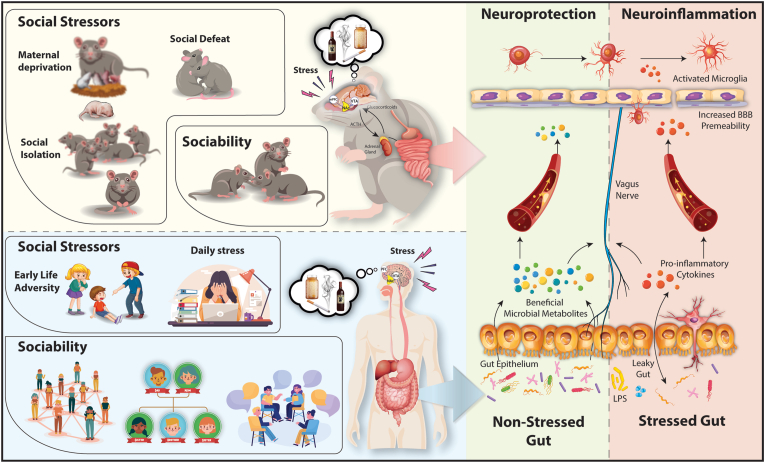
Schematic representation of how social behaviors modulate the stress response and the potential impact of microbiota regulation on neuroinflammation and the activity of the hypothalamic-pituitary-adrenal (HPA) axis, which might contribute to drug-seeking behaviors. Activation of the HPA axis triggered by social stressor might lead to alterations in the microbiota-gut-brain axis. The gut microbiota can stimulate vagal nerve, cytokine production and produce microbial metabolites capable of crossing the blood-brain barrier, reaching brain regions associated with reward circuits and thus influencing drug-seeking behaviors. On the other hand, positive social interactions may attenuate HPA activation, promoting a healthier, less stressed gut environment, which in turns reduces brain inflammatory processes and dampens drug-seeking behaviors.

Just as social stress can be a vulnerability factor for addictions, social interactions can be a resilience factor against addictions (El Rawas et al., 2020; Heilig et al., 2016; Venniro et al., 2018). In this context, positive social interactions and work environments supporting non-drug use, has been shown as effective to prevent substance abuse during adolescence (Valente et al., 2007), supporting the view that social rewards serve as a protective factor, facilitating affective coping promoting resilience against substance abuse (Heilig et al., 2016). This community-reinforcement therapeutic approach has been also back-translated to animal models. In rats, the rewarding effects of social interactions have been determined, with animals preferring to spend more time in the context associated with a positive social interaction (Manduca et al., 2021). These findings indicate that social interactions in a distinct context could be a useful strategy to reduce the incentive salience of drug-associated contextual stimuli (Bregolin et al., 2017; Fritz et al., 2011; Sampedro-Piquero et al., 2019). A recent study, reported that operant social reward prevented drug self-administration in rats that met criteria for addiction (Venniro et al., 2018, 2019), suggesting that at a choice point the value that rats attributed to drugs is low in comparison to non-drug rewards like social interactions. Furthermore, social context can decrease stress responses, demonstrating the contribution of social interactions as a valuable component in treatment of substance use disorders be reducing stress levels (Lemos et al., 2021). From a microbiota point of view, modulations of the microbiota could constitute a valuable therapeutic approach for drug reward related conditions alone or in combination with social factors. Moreover, we have recently shown that depletion of the gut microbiota reduced the reward to non-natural reward such as cocaine and increases social rewards. Moreover, the presence of social stimulus in combination with antibiotics were able to decrease the preference for cocaine, highlighting that two-pronged approach of targeting the gut microbiota and enhancing social behaviors could constitute a value component in reducing harm in drug use by altering the salience effects of cocaine (García-Cabrerizo et al., 2023) (Fig. 2).

## 7 Sex differences in gut microbiota, stress and substance use disorders

There is an increasing body of literature documenting sex differences across various physiological and pathophysiological conditions (Audet, 2019; Miller and Halpern, 2014). The gut microbiota is influenced in part by sex hormones during adolescence and remains stable in adulthood (Baars et al., 2018; Jaggar et al., 2020; Jašarević et al., 2016; Yurkovetskiy et al., 2013). While preclinical studies have demonstrated sex-specific differences in the gut microbiota with significant impacts in immune system development (Markle et al., 2013; Yurkovetskiy et al., 2013), other preclinical studies have suggested that species and strain of mice may exert a stronger effect than sex on microbial composition (Elderman et al., 2018; Kovacs et al., 2011; Org et al., 2016). Differences in gut microbial composition between sexes has been observed across various mouse strains. Males generally exhibited lower microbial diversity than females, with sex explaining 11.6% of the variance in microbiota composition (Elderman et al., 2018). Additionally, sex influenced the expression of immune-related genes in a strain-dependent manner (Elderman et al., 2018). In another study, specific genera such as *Allobaculum, Erwinia* and *Anaeroplasma* were more prevalent in males, while other members of the *Lachnospiraceae* family (*Dorea*, *Coprococcus* and *Ruminococcus*) were more abundant in females in different mouse strains (Org et al., 2016). However, the impact of sex on microbiota composition may vary depending on host genotype, and no clear differences were observed between sexes when examining different mouse strain together (Org et al., 2016). Human studies on sex differences in the gut microbiota have faced challenges due to subtle variations and confounding factors (Borgo et al., 2018; Integrative HMP Research Network Consortium, 2019; Lay et al., 2005; Mueller et al., 2006; Odamaki et al., 2016; Takagi et al., 2019), suggesting that studies with larger cohorts are needed to unmask sex differences.

Sex differences has also been reported in stress-related disorders. In rats, higher basal and stress-induced corticosterone levels have been observed in female compared with males (Viau et al., 2005). Exposure to stressors increases adrenocorticotropic hormone and corticosterone in females, partly due to ovarian hormones enhancing adrenocorticotropic hormone sensitivity and contributing to greater corticosterone release (Atkinson and Waddell, 1997; Carey et al., 1995; Figueiredo et al., 2007). In this line, differences in the neuroendocrine response to stress involve higher corticotrophin-releasing factor expression in the paraventricular nucleus of female rodents (Iwasaki-Sekino et al., 2009; Viau et al., 2005). Gonadal hormones such as estradiol might enhance corticotropin-releasing factor expression in females, while androgens suppress it (Iwasaki-Sekino et al., 2009), with similar effects observed in vasopressin expression (Lund et al., 2004; Lunga and Herbert, 2004). In humans, sex differences in HPA activity are not as clear. While baseline cortisol levels are similar between men and women (Kirschbaum et al., 1999; Uhart et al., 2006), conflicting findings exist regarding stress-induced cortisol levels, possibly due to variations in stressors or participant characteristics (Kudielka et al., 1998; Kudielka and Kirschbaum, 2005; Seeman et al., 2001; Uhart et al., 2006). However, epidemiological data reveal sex differences in disorders exacerbated by stress with women more likely to suffer anxiety, panic disorders, posttraumatic stress disorders and depression (Breslau, 2002; Sheikh et al., 2002; Tolin and Foa, 2006), while men are more likely to suffer substance-related disorders (Bobzean et al., 2014). Nonetheless, women tend to report greater sensitivity to drug effects compared to men, developing substance abuse faster (Becker et al., 2012; Fattore et al., 2014). Understanding sex differences in drug addiction hinges on estrogen's role in enhancing drug-seeking and rewarding effects in females, differentially modulating dopaminergic activity in a sex-specific way (Becker, 2016; Hu and Becker, 2003; Quigley et al., 2021). Preclinical work has indicated sex differences in each phase of the addiction process, with females exhibiting greater vulnerability compared to males (Becker and Koob, 2016). Similarly, in humans, women are more avid drug seekers than man to several drugs and these effects are influenced by estrogens and progesterone (DeVito et al., 2014; Hilz and Lee, 2023; Lynch and Sofuoglu, 2010; Schiller et al., 2012). These findings indicate that neuroactive steroids significantly influence the impact of stress, and addiction susceptibility. However, the role of the microbiota in this vulnerability remains unknown, emphasizing the need for future studies to focus on potential sex differences in the gut microbiota to assess forthcoming mechanisms and identify potential therapies (Peterson et al., 2020).

## 8 Conclusions

The gut microbiota plays a pivotal role in regulating our brain and behavior, with accumulating evidence from both preclinical and clinical studies shedding light on how external factors, such as stress, social interactions, and certain drugs of abuse, can influence the composition of the gut microbiota. In addition, specialized tools and animal models, including GF animals, antibiotic-treated models, and fecal microbial transplants, have enhanced our comprehension of how alterations in the gut microbiota can impact the stress response, modify social behaviors and mediate behavioral responses to drugs of abuse. The studies presented in this review underscore the intricate relationship between the gut microbiota, social stress and addiction. However, most of the focus has been on the bacteriome to date. Recently, it has been shown that stress affects the gut virome and that treatments with a fecal virome transplant could alleviate the behavioral, immune and neurobiological consequences of stress (Ritz et al., 2024b). It is essential to note that causality directly mediated by the gut microbiota has yet to be definitively confirmed. Furthermore, form an intervention perspective, there is a substantial potential for microbiota-based interventions, which hold promise in their ability to potentially modulate social stress and, consequently, may be of substantial interest for addiction treatment. Nevertheless, more focused research in this specific area is needed considering a sex perspective, and deeper understanding of the mechanisms thought which these interventions impact behavior remains imperative.

## CRediT authorship contribution statement

**Rubén García-Cabrerizo:** Writing – review & editing, Writing – original draft, Visualization, Conceptualization. **John F. Cryan:** Writing – review & editing, Writing – original draft, Visualization, Supervision, Conceptualization.

## Declaration of competing interest

The authors declare no potential conflicts of interests.

## Data Availability

No data was used for the research described in the article.
